# Detecting Azole-Antifungal Resistance in *Aspergillus fumigatus* by Pyrosequencing

**DOI:** 10.3390/jof6010012

**Published:** 2020-01-10

**Authors:** Mireille H. van der Torre, Lilyann Novak-Frazer, Riina Rautemaa-Richardson

**Affiliations:** 1 Mycology Reference Centre, Excellence Centre of Medical Mycology (ECMM), Manchester University NHS Foundation Trust-Wythenshawe Hospital, Manchester M23 9LT, UK; mireille.vandertorre-2@manchester.ac.uk (M.H.v.d.T.); Lily.Novak-Frazer@manchester.ac.uk (L.N.-F.); 2 Division of Infection, Immunity and Respiratory Medicine, School of Biological Sciences, NIHR Manchester Biomedical Research Centre (BRC) at the Manchester Academic Health Science Centre, The University of Manchester, Manchester M23 9LT, UK; 3 Department of Infectious Diseases, Manchester University NHS Foundation Trust-Wythenshawe Hospital, Manchester M23 9LT, UK

**Keywords:** *Aspergillus fumigatus*, antifungal drug resistance, *cyp51A*, azole resistance, diagnostics, pyrosequencing

## Abstract

Guidelines on the diagnosis and management of *Aspergillus* disease recommend a multi-test approach including CT scans, culture, fungal biomarker tests, microscopy and fungal PCR. The first-line treatment of confirmed invasive aspergillosis (IA) consists of drugs in the azole family; however, the emergence of azole-resistant isolates has negatively impacted the management of IA. Failure to detect azole-resistance dramatically increases the mortality rates of azole-treated patients. Despite drug susceptibility tests not being routinely performed currently, we suggest including resistance testing whilst diagnosing *Aspergillus* disease. Multiple tools, including DNA sequencing, are available to screen for drug-resistant *Aspergillus* in clinical samples. This is particularly beneficial as a large proportion of IA samples are culture negative, consequently impeding susceptibility testing through conventional methods. Pyrosequencing is a promising in-house DNA sequencing method that can rapidly screen for genetic hotspots associated with antifungal resistance. Pyrosequencing outperforms other susceptibility testing methods due to its fast turnaround time, accurate detection of polymorphisms within critical genes, including simultaneous detection of wild type and mutated sequences, and—most importantly—it is not limited to specific genes nor fungal species. Here we review current diagnostic methods and highlight the potential of pyrosequencing to aid in a diagnosis complete with a resistance profile to improve clinical outcomes.

## 1. Diagnosis of Aspergillosis

Aspergillosis is a fungal infection typically acquired via the inhalation of *Aspergillus* spores. It is most often caused by *A. fumigatus* and its various forms affect millions of people worldwide [1]. In immunocompetent hosts, these moulds cause a localized infection mainly in the lungs or paranasal sinuses. In immunocompromised individuals, the inhalation of spores can lead to a life-threatening invasive respiratory infection and dissemination to other organs. This invasive manifestation of aspergillosis (IA) is most life-threatening, with mortality rates varying between 50% when treated promptly and above 80% when the treatment is delayed [1,2,3,4,5].

When suspecting IA, the guidelines recommend a computed tomography (CT) scan to detect pulmonary infiltrates [6,7,8,9]. Nodules surrounded by a ground-glass attenuation (halo sign) and pleural effusions are classical CT findings of IA. Serum and bronchoalveolar lavage (BAL) samples should be sent for galactomannan (GM) testing and for PCR targeting *Aspergillus*-specific 18S rDNA [10,11]. Parallel testing for serum β-1-3-d-glucan (BDG) improves specific detection. When used in combination, these tests provide adequate sensitivity and specificity to guide antifungal treatment [6,8,12]. Direct microscopy of BAL or biopsy materials using fluorescent dyes can rapidly detect an invasive mould infection, but cannot provide a definitive diagnosis. Therefore, samples should also be sent for fungal culture, identification and susceptibility testing. However, culture is a slow and insensitive method, although its sensitivity can be improved by increasing the culture volume [13,14]. The EQUAL Aspergillosis Score 2018 is a stewardship tool summarising the key aspects of the ESCMID IA guidelines. [15].

A neutropenic haematology patient presenting with fever, chest pain and a cough, and suspected of IA when they are not responding to broad-spectrum antibiotic treatment, is a common clinical scenario. They typically have abnormal CT findings suggestive of IA; their serum and/or BAL is positive for one or more of the biomarkers, but the culture is reported with no fungal growth. The patient is started on first-line empiric treatment with voriconazole or isavuconazole [6,8]. With the rise of azole-resistant *A. fumigatus* strains, it is not rare that the patient fails to respond to the treatment due to antifungal resistance [16,17,18,19,20,21,22]. This is seen in patients who have been on azole prophylaxis (e.g., posaconazole) but increasingly also in azole-naïve patients. Annual surveys in five academic hospitals in the Netherlands showed that azole resistance in *A. fumigatus* has doubled from 2014 to 2018 [23,24]. Similar data have been reported worldwide [25,26,27,28,29,30,31,32,33,34,35], and an international resistance surveillance group has been set up by ISHAM/ECMM to capture more information on azole resistance worldwide [36,37].

Currently, the only method recommended by the guidelines to detect azole resistance in patients suspected of having an infection caused by azole-resistant *A. fumigatus* is susceptibility testing by culture [6,8,38,39]. However, more than half of BAL cultures are reported with no fungal growth and IA diagnosis is made based on radiology, fungal biomarker and molecular test results which do not provide information on resistance [40,41,42,43]. In these cases, failure to respond to treatment is often the only evidence of resistance to the given drugs. On the other hand, even in the case of a positive culture and access to susceptibility testing or resistance screening, the turnaround times for these culture-based methods are long, and the delivery of results in clinically useful time frame is challenging. Particularly in smaller centres, where susceptibility testing is not available on site, this timeline often exceeds one week [39,44]. 

The outcome of IA depends on the early initiation of effective treatment. This relies on rapid detection of antifungal resistance. Failure to detect azole resistance leads to significantly increased mortality rates in azole-treated patients [45]. Here, we describe the available methods for the detection of azole resistance in *A. fumigatus*, with a focus on a novel pyrosequencing-based method using the *A. fumigatus cyp51A* target. This method can detect any polymorphisms in critical genes, provide epidemiological data on azole resistance and also provide a quick and reliable diagnosis in culture-negative cases.

## 2. Azole Resistance

Mutations in genes involved in the *A. fumigatus* ergosterol biosynthetic pathway, including *hmg1, erg6, cyp51A* and *cyp51B* among others, are described as having a role in azole resistance [26,46,47,48,49,50,51]. The means for testing non-*cyp51A*-mediated mechanisms of resistance lag far behind and therefore, for the purpose of this discussion, we concentrate on *cyp51A*. Azole-resistant strains harbour genes with specific point mutations in combination or absence of tandem repeats in the promoter region. *Aspergillus* species are intrinsically resistant to fluconazole and ketoconazole due to a naturally occurring point mutation in *cyp51A,* encoding lanosterol 14-α-sterol demethylase [52]. Polymorphisms in *cyp51A* are frequently described as the main resistance mechanism arising from long-term azole use, associated with chronic aspergillosis [27,28,53]. Alternatively, the most common pan-azole resistance mechanism in *A. fumigatus cyp51A* was found to be a combination of a 34-bp long tandem repeat (TR) in the promoter region and a leucine-to-histidine change at codon 98, in short TR34/L98H [54,55,56,57,58,59]. The TR46/Y121F/T289A is a less widespread but emerging azole resistance mutation, which was first reported in the Netherlands in 2009 [60]. Both resistance mechanisms are regularly recovered from environmental isolates worldwide [31,61,62,63,64,65,66,67,68,69,70,71], and therefore are likely to have developed resistance from the substantial use of environmental azole fungicides [31,55,69,72,73,74,75,76,77,78]. In 2017, the first azole-resistant *A. fumigatus cyp51A* TR46/Y121F/T289A mutant was isolated in the UK from a patient that had no prior history of azole antifungal use, suggesting resistance must have been acquired through the environment [79]. The global prevalence of environmental azole-resistant *A. fumigatus* strains is a major concern for all, but particularly susceptible patients.

Numerous single point mutations in *A. fumigatus cyp51A* lead to resistance, where the most common include amino acid substitutions of glycine at codon 54 (G54), proline at codon 216 (P216), phenylalanine at codon 219 (F219), methionine at codon 220 (M220), and glycine at codon 448 (G448) (Table 1) ([18,26,54,80,81,82,83,84,85,86,87,88,89,90,91,92,93] and reviewed in [94]). Other resistance mechanisms are described to be a combination of point mutations or the less common TR53 [89,95,96,97,98,99,100,101,102]. 

## 3. Molecular Techniques for Discerning *cyp51A* Resistance Polymorphisms

Molecular tests for the detection of azole-resistant *cyp51A* genotypes of *A. fumigatus* in clinical specimens have been developed over the past 20 years. Numerous in-house assays ranging from nested PCR, mixed-format and real-time PCR approaches have been developed and tested [103,104,105,106,107,108,109,110]. Other molecular approaches (reviewed in [105]) include the analysis of high resolution melt curves [111,112]. However, despite all this work, only three commercial kits are currently available (AsperGenius, PathoNostics, Maastricht, Netherlands, MycoGENIE, Ademtech, Pessac, France and Fungiplex^®^ Aspergillus Azole-R IVD PCR, Bruker Daltonik GmbH, Bremen, Germany), attesting to the difficulty of developing robust molecular assays [113]. All three commercial real-time PCRs (RT-PCR) are designed to identify the promoter region insertion polymorphisms TR34/L98H and TR46/Y121F/T289A and specific alleles [109,114,115,116,117,118,119]. Approaches targeting polymorphisms in the *cyp51A* open reading frame (ORF, e.g., G54, M220, G138, G448) [120,121,122] have lagged behind.

Pyrosequencing is an alternative molecular method, which can be used to screen for all known polymorphisms [123]. This DNA sequencing technique has recently been optimised to screen clinical respiratory samples and *A. fumigatus* isolates for both insertion and ORF *cyp51A*-associated polymorphisms [124]. The efficacy of pyrosequencing to detect resistance in respiratory samples from patients with chronic pulmonary aspergillosis (CPA) was demonstrated in a recent audit of UK National Aspergillosis Centre (NAC) cases [125]. Resistance was identified in almost a quarter of culture negative samples, translating to a significant number of resistant cases that would have been missed had pyrosequencing not been used.

## 4. The Pyrosequencing Method

**Figure 1:**
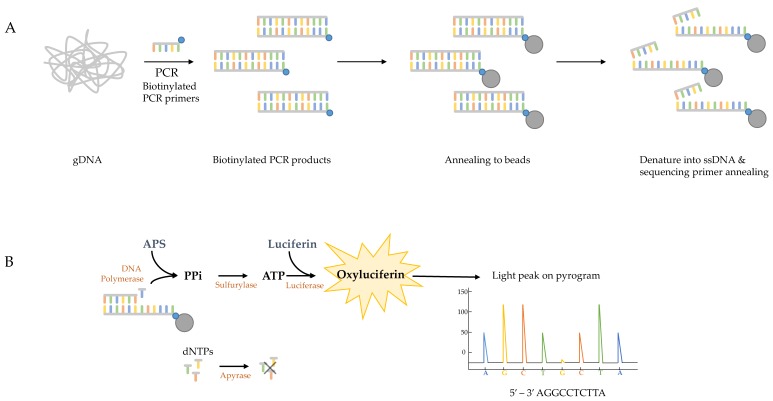
Schematic overview of the pyrosequencing method. (**A**) Genomic DNA (gDNA) of clinical samples or fungal isolates are used as template in the initial PCR with biotinylated (blue dots) primers. The biotinylated PCR products are immobilized by annealing to streptavidin-sepharose beads (grey dots). The DNA strands are then separated allowing the sequencing primer to anneal to the ssDNA templates. (**B**) The four enzymes (polymerase, sylfurylase, luciferase and apyrase) and two substrates (adenosine 5’ phosphosulfate (APS) and luciferin) promote the production of light after nucleotide incorporation in every cycle, resulting in light peaks on the pyrogram that are representative of the DNA sequence. The excess nucleotides are degraded after each cycle by apyrase and the reaction solution is rejuvenated for the incorporation of the next nucleotide.

**Figure 2:**
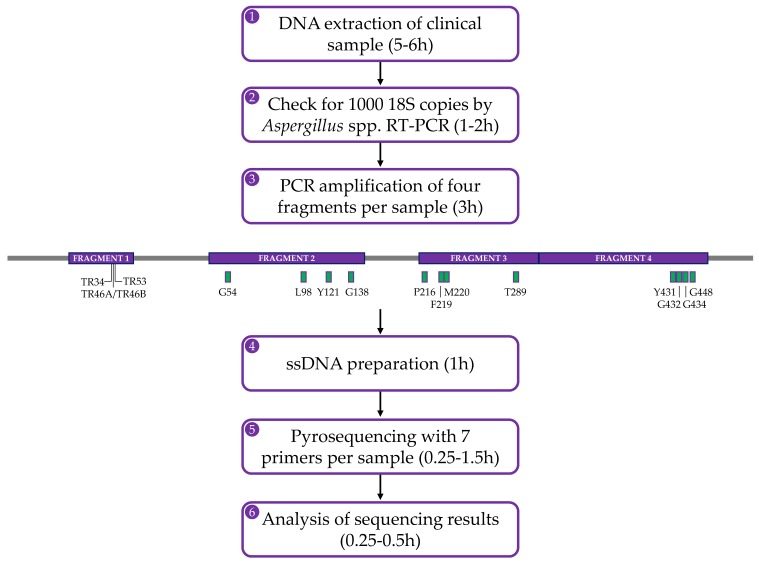
Flowchart of the in-house pyrosequencing-based method showing the steps that are involved to complete one pyrosequencing experiment. The initial steps (1–3) to enable pyrosequencing require the detection of ample *A. fumigatus* genomic DNA. The *cyp51A* gene, including 550 bases upstream and 58 bases downstream of the ORF, is targeted as four fragments (from 235–427 bp in length) by endpoint PCR using biotinylated primers as shown in purple in the gene diagram (modified with SnapGene software from GSL Biotech; available at snapgene.com). TR sites are specified in fragment 1 and SNPs associated with resistance (green boxes) are indicated in fragments 2–4. Following confirmation of successful amplification, PCR products are processed (steps 4–5) using the PyroMark Q24 Advanced instrument, kit and accessories (Qiagen, GmBH, Hilden, Germany). Each fragment comprises at least two hotspots (or insertion in the case of the 5′ upstream fragment) which is sequenced individually. Pyrograms are analysed (step 6) manually against a reference *A. fumigatus* strain sequence.

The pyrosequencing method is based on the ‘sequencing-by-synthesis’ principle and is widely used to detect epigenetic modifications in malignant cells and microbes but also to discover biomarkers [126,127]. The method relies on the detection of light that is released during nucleotide incorporation into the amplifying DNA (Figure 1). The first step in the assay for the detection of azole-resistant *cyp51A* genotypes of *A. fumigatus*, is to obtain the template DNA by PCR amplification of target gene fragments using genomic *A. fumigatus* DNA or patient respiratory samples. The sense strand must be labelled with 5′ biotin as biotinylated PCR products will bind to the streptavidin–sepharose beads and—since only one strand carries biotin—this promotes the generation of ssDNA template [128,129]. Once the ssDNA fragments are immobilized, the sequencing primer needs to be annealed to the template ssDNA and the pyrosequencing reaction can begin (Figure 1A). During the pyrosequencing reaction, pyrophosphate is released when a nucleotide is incorporated to the growing dsDNA product and is converted by sulfurolyase into ATP, which is then utilized by luciferase to produce light (Figure 1B). Nucleotides are added sequentially to the enzymes- and substrates mixture and, therefore, the sequence can be read as the reaction proceeds. The light peak on the pyrogram is proportional to the number of incorporated nucleotides. After every cycle, the enzyme apyrase degrades the excess nucleotides and regenerates the reaction solution for the next nucleotide [130], which can be added in a known order corresponding to the nucleotide sequence of the region or in an arbitrary, repeated order. Single point mutations can be identified by comparing the pyrogram of the synthesised DNA with that of the reference DNA. To summarise, completing one pyrosequencing run includes DNA extraction of the clinical sample, followed by PCR amplification of the four target gene fragments and ends with the pyrosequencing reactions of biotinylated PCR products (Figure 2). 

This pyrosequencing assay generates reads of up to 150 bp per reaction and provides a large number of sequence reads in a single run, resulting in significant sampling depth. This allows the detection of not only the most numerous specific *A. fumigatus cyp51A* sequences, but also of the lower-abundance reads, which is especially important in processing clinical samples where there is a potential mixture of genomes from human tissue, fungal hyphae of different *A. fumigatus* genotypes, other fungi and other microorganisms. An advantage of this method is that PCR amplicons obtained by endpoint PCR do not need to be purified prior to setting up the pyrosequencing reactions, only checked for successful amplification by agarose gel electrophoresis. Furthermore, the limit of detection is similar to that of RT-PCR-based assays. In practice, clinical samples positive by *Aspergillus* spp. RT-PCR (>1000 18S copies equivalent to approximately 50 genomes) routinely yield successful pyrograms.

It is common that samples from patients with CPA on azole therapy remain negative in culture weeks after the onset of clinical deterioration later shown to be due to azole resistance. Therefore, a sensitive assay such as pyrosequencing can provide clinically useful results and have a significant impact on patient outcomes [14]. The in-house pyrosequencing-based assay is easily implemented within a molecular-based workflow and can be used to screen clinical samples directly within 11–14 h, from start (extraction) to finish (pyrogram analysis). This rapid turnaround time can help achieve a prompt diagnosis, resulting in more effective treatment and consequently reduce mortality and morbidity due to *Aspergillus* infections. The *cyp51A* screening method can also be utilised to monitor and report the local prevalence of *cyp51A* genotypes. In practice, we assay patients who are suspected of starting to fail therapy and follow up every time they produce a PCR-positive sample, something already shown to be important for regular monitoring of patient well-being [54].

A significant advantage of the pyrosequencing assay over RT-PCR strategies is that the method can be adapted to any gene or genomic region of interest. The current *A. fumigatus* PCR-based resistance assays do not reveal the less common or potential mutations involved in azole resistance. In Manchester, 43% of the azole-resistant patient isolates were reported to be non-*cyp51A* related [26] and numerous studies have revealed that azole resistance is most certainly due to other genes [46,47,48,49,50,51,131,132,133,134]. Besides gene mutations in the ergosterol biosynthesis pathway, other non-*cyp51A*-mediated resistance mechanisms involve the overexpression of drug efflux transporters such as ABC- and MFS-transporters [96,135,136,137]. Therefore, sequencing entire target genes can be more informative and can be done using the classical Sanger method or Next-Generation Sequencing (NGS). A small study of four azole-resistant *A. fumigatus* isolates using NGS revealed non-*cyp51A* polymorphisms including point mutations in *SrbA, Mdr1, abcE, ERG3* and *ERG24* and amino acid substitutions in *HMG1, ERG3* and *ERG24* [48]. Although most laboratories do not yet have the capacity to perform NGS in-house, pyrosequencing is relatively cost-effective because the approach is targeted to a specific region or ‘hot spot’ and requires less equipment (a thermal cycler for endpoint PCR and the PyroMark Q24 Advanced instrument).

Pyrosequencing is already an established technology for exploratory and testing in a broad range of disciplines, particularly those in which culture dependent methods are limited by insensitivity [138,139,140,141,142]. Moreover, in comparison to Sanger sequencing, pyrosequencing is favoured due to factors such as assay sensitivity, specificity, limit of detection, detection of rare and mixed mutations, turnaround time and costs [140,143]. Regular monitoring for resistance by pyrosequencing offers the potential for early appropriate drug therapy leading to better clinical outcomes, improved antifungal stewardship and cost savings. At the UK NAC, the annual cost of azole therapy was £2,049,900 in 2018/19 for 543 patients, a cost of approximately £3800 per patient to the UK National Health Service (NHS) [144]. A prevalence of approximately 5.3% azole resistance (indicated by Band 3 classification) indicates that, in addition to the patient benefit achieved by targeted drug therapy, a considerable cost saving could be made by avoiding unnecessary or inappropriate drug administration if samples were screened for resistance mechanisms parallel to standard diagnostic tests. One study found that the median time in which wildtype isolates develop azole resistance can be as short as 4 months in patients treated with azoles [54]. It is hoped that by screening patients with chronic forms of aspergillosis, who are on long term azole-antifungal therapy and at risk of acquiring azole resistance [145,146], we can capture drug resistance early.

## 5. Conclusions

In summary, the incidence of azole-resistant *Aspergillus* infections and the associated poor clinical outcomes make screening for resistance mechanisms crucial in the management of aspergillosis. A combination of tests is needed to assure effective treatment and allow antifungal stewardship. Commercially available PCR assays can be useful for time-pressing samples in smaller clinical laboratories, but the range of polymorphisms they are designed to detect is very limited. Pyrosequencing is a promising in-house DNA sequencing method that can be used to screen for genetic hotspots associated with antifungal resistance rapidly and is not limited to specific genes nor fungal species. The rise of antifungal resistance is ongoing, and new mutations associated with resistance might be missed without a sequence-based approach. In addition to the analysis of *cyp51A*, this method can easily be exploited to look at other genes that play a role in either azole resistance or resistance against other antifungal drugs. Laboratories that do not have real-time thermocyclers or the capacity for in-house sequencing should consider sending their samples for sequencing to either mycology reference centres or companies providing sequencing services. Rapid and reliable diagnosis is important for survival of IA patients and screening clinically relevant samples will provide important epidemiological data on *A. fumigatus*. 

## Figures and Tables

**Table 1:** Overview of the known and emerging cyp51A-associated azole resistance mechanisms.

*cyp51A* Polymorphism	Reported Azole Resistance	Comment	Reference(s)
TR34/L98H	pan-azole	Most common resistance mechanism. Only displays azole-resistant phenotype in combination with L98H substitution.	[27,28,57,58,59,61,63]
TR46/Y121F/T289A	voriconazole, posaconazole	Variable susceptibility to itraconazole. The single point mutation T289A does not result in azole resistance, whereas the single mutation TR46 has a slight reduced pan-azole susceptibility.	[56,59,60,67,69,79]
TR53	itraconazole, voriconazole	Not yet reported in combination with single point mutations	[95]
TR120/F46Y/M172V/E427K	pan-azole	A clinical case of infection with azole-resistance acquired during long-term azole treatment	[102]
N22	itraconazole	Voriconazole and posaconazole susceptibilities not reported	[80,81]
G54	itraconazole, posaconazole	-	[27,54,80,81,82,83,91,92]
Y121	voriconazole	-	[56,97]
G138	pan-azole	-	[27,86,89,99]
Q141	pan-azole	Reported in combination with TR34/L98H	[98,101]
H147	pan-azole	Reported in combination with G448	[27]
P216	itraconazole, posaconazole	-	[27,54,92]
F219	itraconazole, posaconazole	-	[54,85]
M220	itraconazole, posaconazole	Variable susceptibility to voriconazole	[26,27,80,81,84,92]
M236	itraconazole	-	[81]
A284	pan-azole	The single point mutation results in reduced pan-azole susceptibility. Pan-azole resistance is reported in combination with TR34/L98H.	[26,98]
S297	pan-azole	Reported only in combination with TR34/L98H	[57,58,100]
P394	itraconazole	Voriconazole and posaconazole susceptibilities not reported	[80]
Y431	pan-azole	-	[27,83,86,89]
G432	itraconazole, voriconazole	-	[90,100]
G434	pan-azole	-	[27,86,89]
T440	itraconazole	Voriconazole and posaconazole susceptibilities not reported	[80,81]
G448	itraconazole	Variable susceptibility to voriconazole and posiconazole	[27,83,86,87,88,93]
Y491	itraconazole	Voriconazole and posaconazole susceptibilities not reported	[80,81]
F495	pan-azole	Reported only in combination with TR34/L98H	[58,100]
